# Supplementary material for: Different carotenoid conformations have distinct functions in light-harvesting regulation in plants
Source: Nat Commun. 2017 Dec 8;8:1994. doi: 10.1038/s41467-017-02239-z (PMC5722816; doi:10.1038/s41467-017-02239-z)
Supplement: Supplementary file 1 — Supplementary Information [file 41467_2017_2239_MOESM1_ESM.pdf]

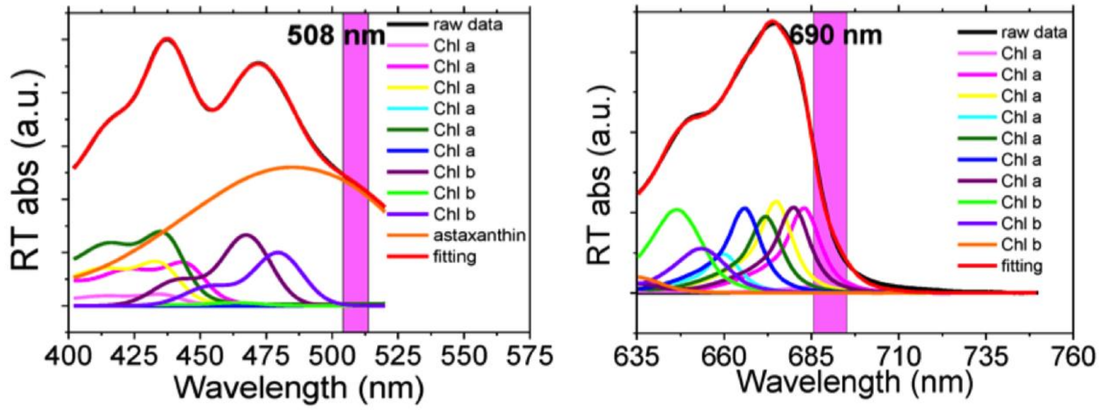

Supplementary Figure 1. Deconvolution in single pigment forms of the LHC-Asta absorption spectrum. Spectral deconvolution with the spectra of individual pigments of the room-temperature absorption spectra in the Soret (left) and Q_X/Q_Y region (right) of LHC-Asta, as in Ref. [1]. The excitation wavelengths used for the transient absorption experiments are indicated by the pink rectangle in the plot centered at the excitation wavelength ±5 nm, to represent the laser pulse width.

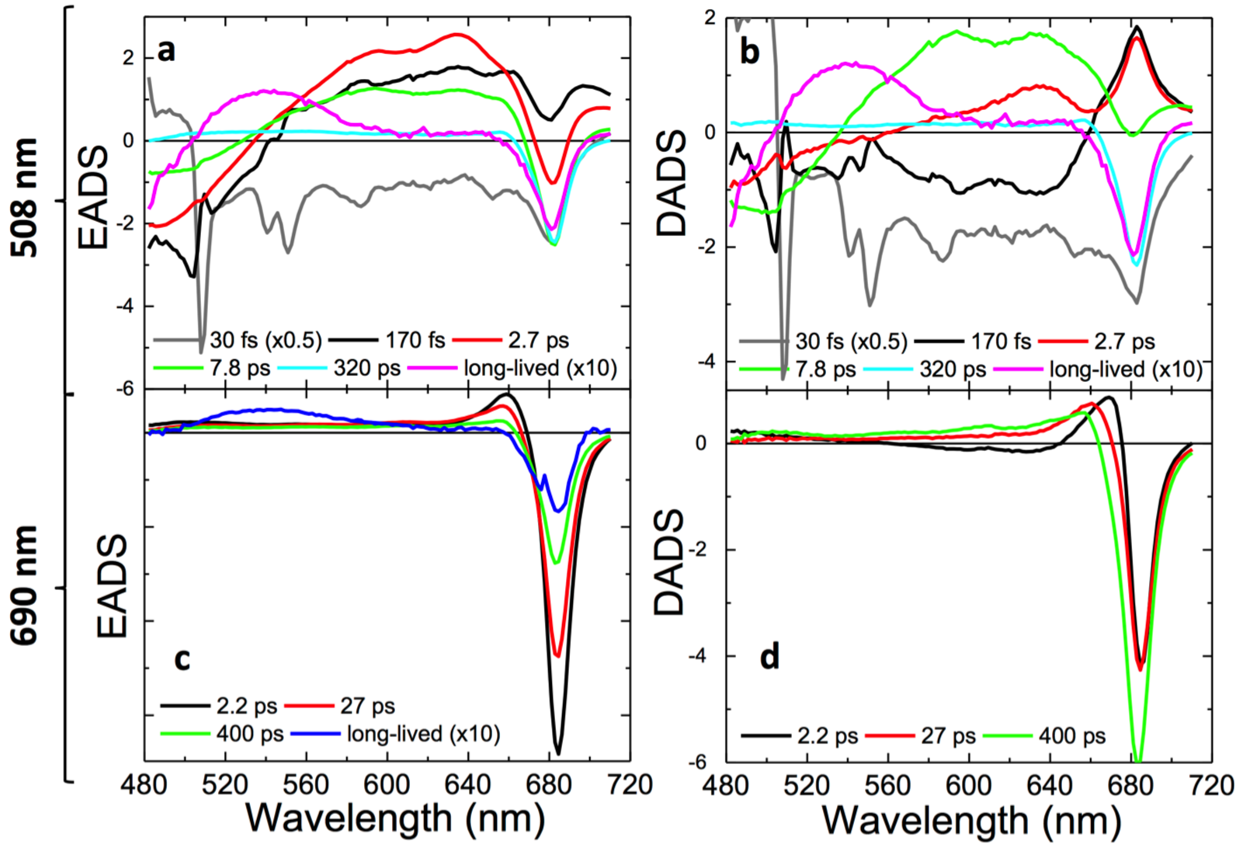

Supplementary Figure 2. DADS and EADS estimated from the global analysis applied to Cars and Chls excitations. (a, c) EADS and **(b, d)** DADS estimated for **(a, b)** Car excitation (508 nm) and **(c, d)** Chl excitation (690 nm). In **(a, b)**, the different lifetimes retrieved from the global analysis, as based on the assignments described in the main text, are associated to the following processes: (i) 30 fs – coherent artifact, decay of the Car S2 state and rise of the Car hot S1 state, (ii) 170 fs – vibrational relaxation of the Car hot S1 to the Car S1 state and transfer from Car hot S1 to Chls (680nm and 650 nm

shoulder), (iii) 2.7 ps – decay of the Car S1 state and transfer from Car S1 to Chls, (iv) 7.8 ps – decay of a Car dark state different from S1 (assigned to S*, as explained in the main text), (v) 320 ps – decay of the Chls, (vi) long-lived component – rise of the Car triplets and possibly decay of the Chls. Similarly, in **(c, d)**: (i) 2.2 ps – transfer from superradiant red Chls to higher energy Chls and transfer from Chls to a different species with Car-like ESA, which we attribute to an unknown Car state "S_q", (ii) 27 ps and (iii) 400 ps – decay of Chls and excitation energy transfer from Chls to a different species assigned to Sq (see main text), (iv) long-lived component – rise of the Car triplets. In **(d)** only the first three components are shown for clarity, as in Fig. 2 of the main manuscript. In **(a)** and (**b**) the grey EADS and DADS are associated to ultrafast (<100 fs) and the magenta (blue in (**c**)) to long-lived (>ns) species. These have been scaled for clarity, as indicated in the legend.

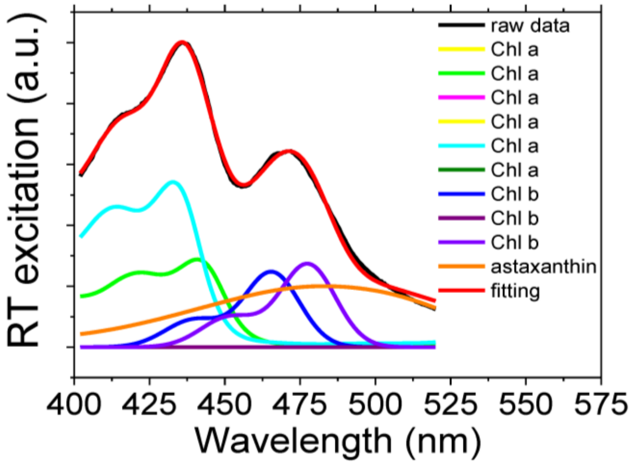

Supplementary Figure 3. Deconvolution in single pigment forms of the LHC-Asta excitation spectrum, with Chl detection. The room-temperature excitation spectrum of LHC-Asta detected at 690 nm (black) is fitted with absorption spectra of Chl *a* and *b* and astaxanthin, similarly to what was previously reported in Ref. [1]. The excitation spectrum was recorded at low OD (0.05 cm^{-1}). The presence of astaxanthin in the excitation spectrum demonstrates energy transfer from astaxanthin to Chl. The transfer efficiency is low by comparison with the absorption spectrum shown in Supplementary Figure 1a.

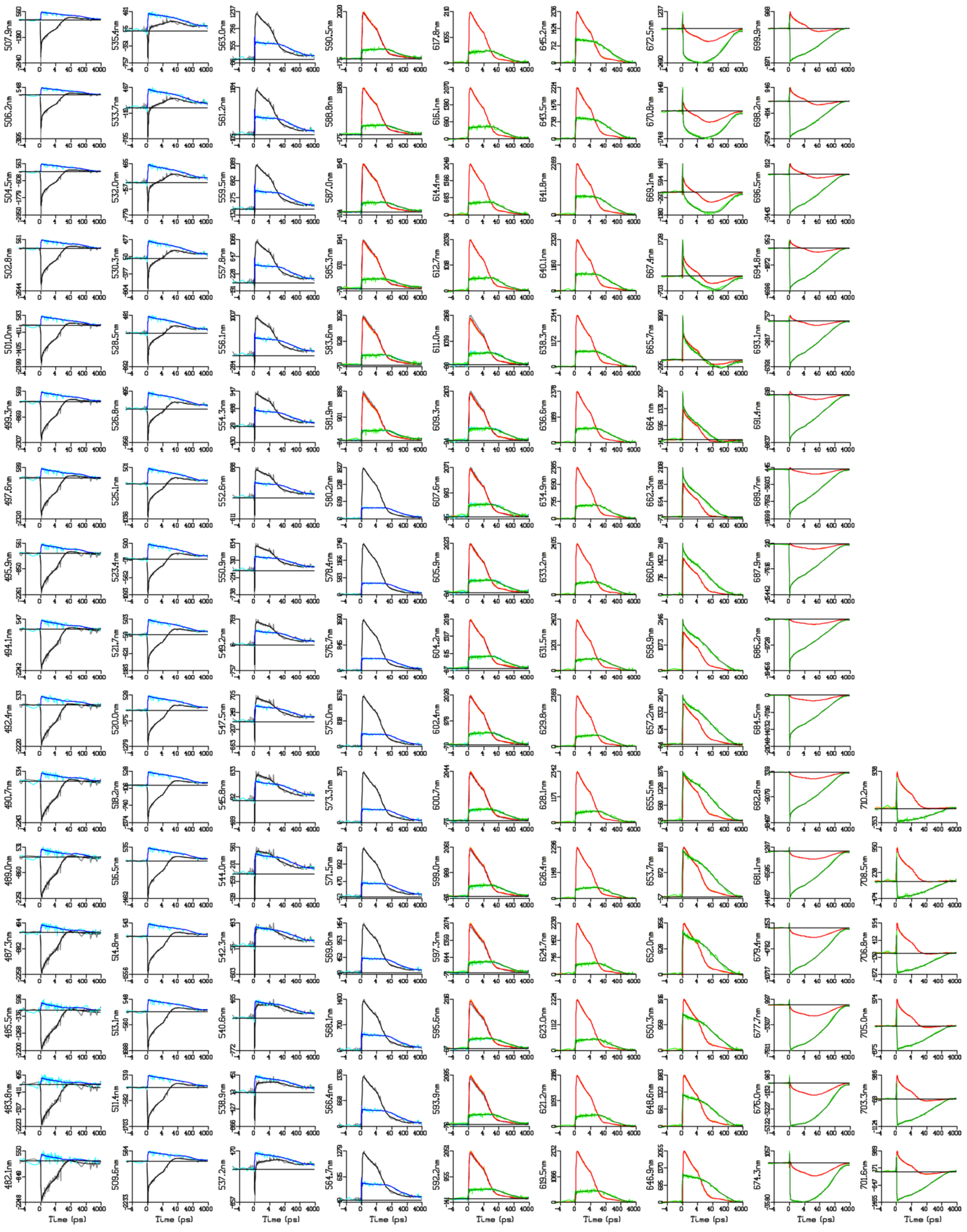

Supplementary Figure 4. Time traces of raw data, light colors, in μOD, and fit, dark colors. The wavelength is indicated in the ordinate label of each panel. For the 508 nm excitation, the traces are shown in grey/black and orange/red, for the 690 nm excitation in cyan/blue and light green/dark green. Note that the time axis is linear until 4 ps after the maximum of the IRF, and logarithmic thereafter.

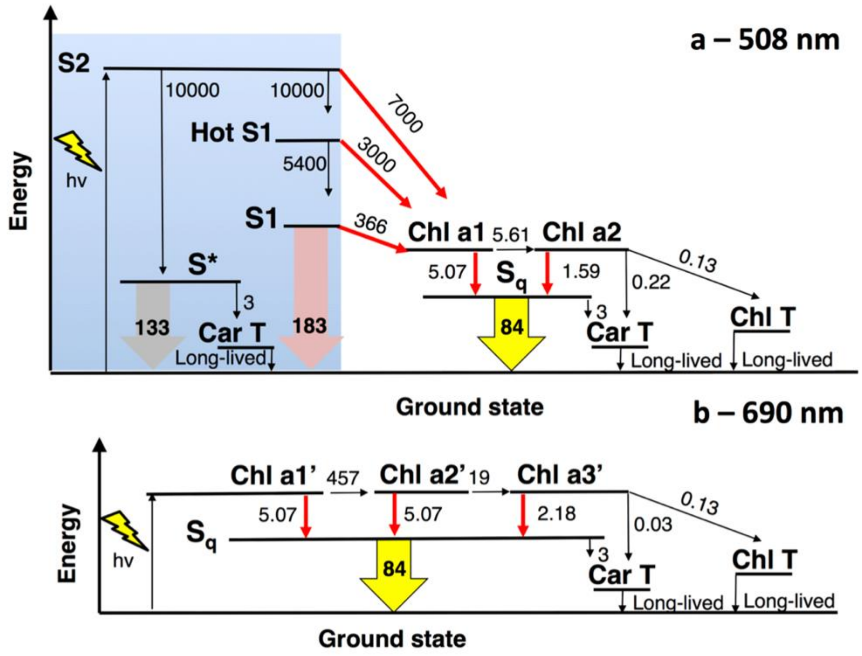

Supplementary Figure 5. Complete model used for the target analysis of the datasets collected upon Car and Chl excitations. In **(a)** and **(b)**, the complete reaction schemes used respectively for the 508 nm and 690 nm excitation wavelengths are presented, including the associated rates in ns^{-1}.

The models are based on the following assumptions: (i) Two pools of astaxanthin, unconnected and connected ones, are present in LHC-Asta in equal amounts as indicated by the biochemical analysis (see main text). In the figure, the unconnected pool follows the scheme enclosed in the blue square in **(a)**. To limit the degrees of freedom of the fit, the only difference between connected and unconnected astaxanthin is the presence/absence of EET to Chl (internal rates and SADS are assumed identical). (ii) The decay of astaxanthin takes place via the "hot" S1, S1, S* and triplet states. (iii) EET from the connected astaxanthin to Chl occurs from the S2, "hot" S1, and S1 states as observed in the global analysis (see main text and Supplementary Figure 2). (iv) A state, called S_q, serves as a de-excitation pathway for Chls. To limit the number of free parameters, the spectrum of S_q below 520 nm has been linked for S1, S* and S_q, similar to what was done previously for other LHC systems[2,3]. Above 650 nm, the S_q SADS is constrained to be zero because the estimate of the small S_q signal is imprecise in the presence of the spectral evolution of the strong Chl Q_y-bands. Removal of these constraints does not improve the fit nor does it change the spectral shape of the ESA of S_q. The estimated SADS is shown in Supplementary Figure 6. (v) Astaxanthin triplets (Car T) are formed directly via S* (and via Chls on longer timescales), as previously shown in the case of spheroidene and rhodopin glucoside bound to bacterial LHC (LH2)[2,3]. Additionally, Car T are generated via S_q, given the similarity between this state and S* (see main text).

Given the time resolution of our experiment (see Methods), all rates corresponding to ultrafast timescales (<<100 fs) and the rates corresponding to long-lived species (>>ns) have been fixed. The Chl T species represents both Chl triplets and the small fraction of long-lived Chls also detected via time-resolved fluorescence (Supplementary Table 1). The SADS presented in Fig. 3.a and 3.c in the main text have been estimated by fitting simultaneously 4 datasets: one obtained upon excitation at 508 nm with an energy per pulse equal to 5 nJ, and three obtained upon excitation at 690 nm at different energies (5, 10 and 15 nJ). For the 690 nm-excitation, a model accounting for annihilation effects was added to the target scheme as explained in the caption of Supplementary Table 2. The SADS and decay rate of S_q were constrained to be the same in the four datasets.

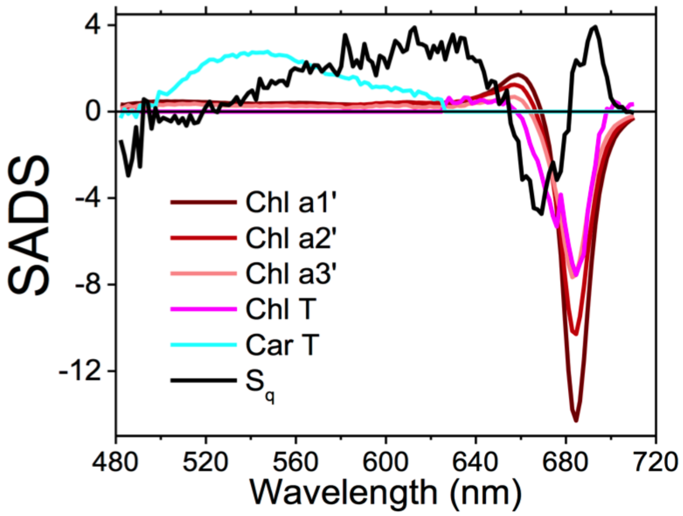

Supplementary Figure 6. SADS estimated for the dataset collected upon Chl excitation without spectral constraints on S_q. SADS estimated via a model identical to the one presented in Figure 3d and Supplementary Figure 5b where, however, the spectral constraints on the S_q compartment, used to minimize the number of free parameters as explained in the caption of Supplementary Figure 5, have not been used. The estimated S_q SADS shows the same ESA as in Fig. 3c. It is, however, more noisy in the bleach region, where the probe light is noisy. In the Chl-Q_y region, the spectrum shows a bandshift-like shape. This feature might originate either from an evolution in the Chl pool simultaneous to the one of S_q and, therefore, captured by this SADS in the analysis, or from excitonic coupling between Cars and Chls[4].

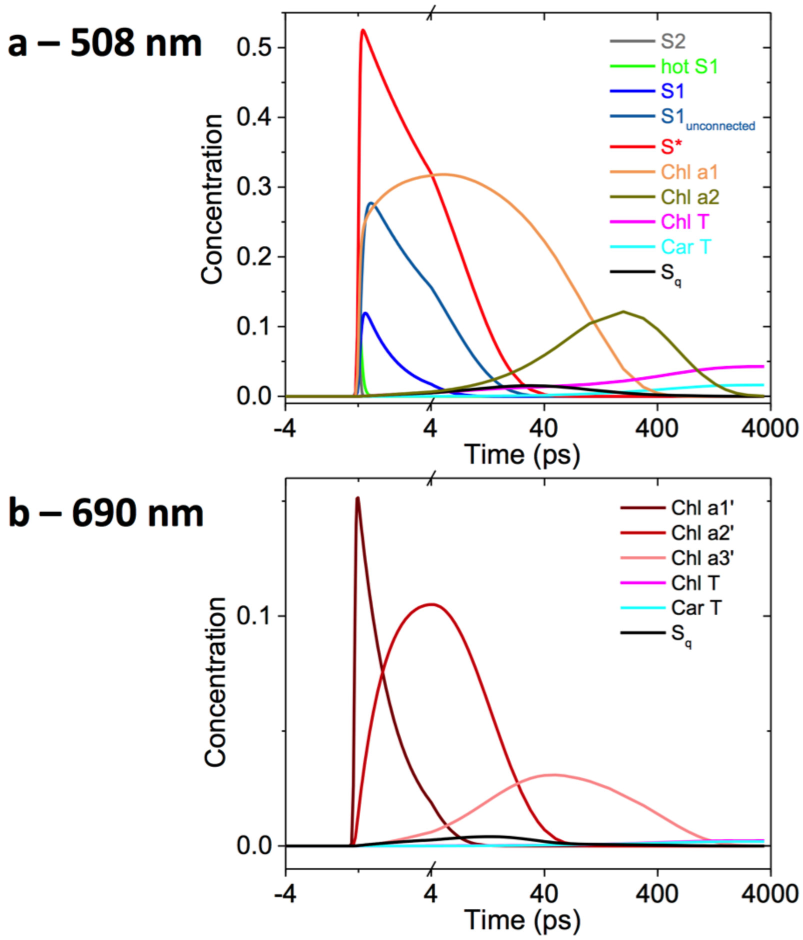

Supplementary Figure 7. Concentrations of the different species estimated via target analysis of the datasets collected upon selective excitation of Cars and Chls. (a) Concentration profiles for the different components according to the model presented in Figure 3b and Supplementary Figure 5a (508 nm-excitation wavelength). The subscript "unconnected" refer to the astaxanthin considered unconnected (Supplementary Figure 5a). The concentrations of S2, hot S1 of the unconnected astaxanthin are not shown here. The concentration of S* is the sum of the ones of both connected and unconnected astaxanthin. **(b)** Concentration profiles for the different components according to the model presented in Figure 3d and Supplementary Figure 5b (690 nm-excitation wavelength). Note that the time axis is linear until 4 ps and logarithmic thereafter.

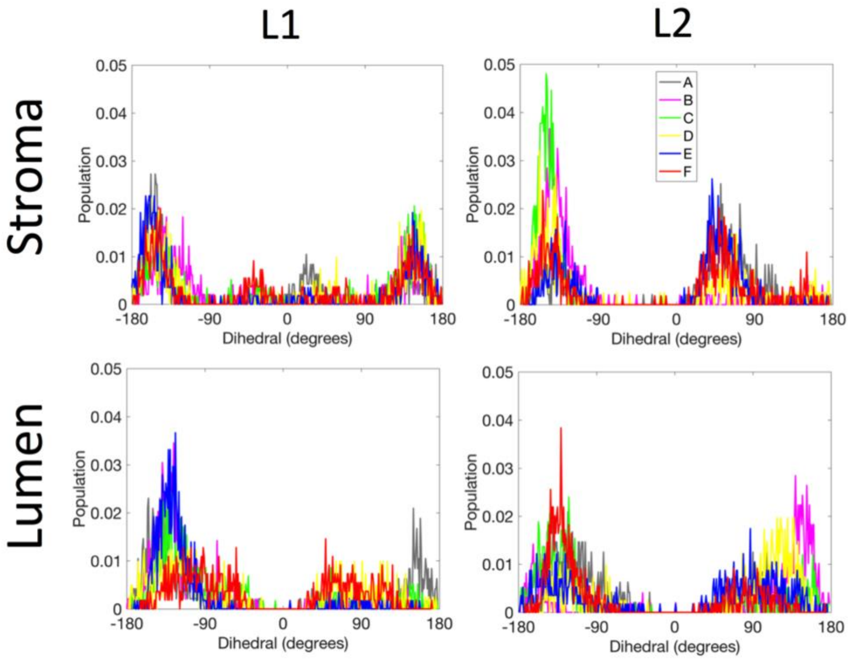

Supplementary Figure 8. Conformational flexibility of the Cars' end rings in LHC-Asta. The dihedral distributions for the two end-rings of astaxanthin bound to site L1 and L2 of LHCII were computed over 6 independent MD simulations per each complex (simulation A to F, see Methods), discarding the first 400 ns of simulation per each run as in Fig. 5 of the main manuscript. In the top panel and bottom panel, the dihedrals of the ring located respectively at the stromal and lumenal are reported. The color scheme for all the plots follows the legend inserted in the plot associated to the L2 site, stromal side (upper right panel).

	τ_1 (A_1)	τ_2 (A_2)	τ_3 (A_3)	$T_{average}$
**LHCII-WT**	0.3 ns (16%)	1.9 ns (36%)	3.7 ns (48%)	2.5 ns
**LHC-Asta**	0.3 ns (60%)	1.0 ns (31%)	2.7 ns (9%)	0.7 ns

Supplementary Table 1. Fluorescence lifetimes of LHCII-WT and LHC-Asta. The results of the global analysis of the kinetics acquired via time-correlated single photon counting (TCSPC) acquisition are listed in the table. We acquired the emission decay at the wavelength corresponding to the LHCII-WT and LHC-Asta maxima (680 nm) upon 470 nm-excitation (chlorophyll b and astaxanthin region). The fluorescence yield is proportional to the average fluorescence lifetime. Therefore the fluorescence yield of LHC-Asta relative to LHCII-WT is 0.7/2.5=0.28.

Power	LHC-Asta$_{_NA}$ (%)	LHC-Asta$_{_A}$ (%)
**5 nJ**	**99**	**1**
**10 nJ**	**95**	**5**
**15 nJ**	**86**	**14**

Supplementary Table 2. Annihilation contribution to the Chl excitation. To quantify the possible contribution of singlet-singlet or singlet-triplet annihilation to the kinetics recorded upon 690 nm excitation the model presented in the main text has been applied to 3 datasets collected at increasing powers (see Table). The fraction of LHC-Asta without annihilation (LHC-Asta$_{_NA}$) follows the kinetic schemes of Fig. 3d and Supplementary Figure 5b. The fraction with annihilation (LHC-Asta$_{_A}$) follows a modified scheme, where additional decay rates to the ground state of 56 ns^{-1} have been added to the Chl a1', a2' compartments.

Stroma	L1		L2		N1	
Simulation	hydroxyl	keto	hydroxyl	keto	hydroxyl	keto
A	-	-	40	42	-	-
B	-	-	51	7	-	-
C	-	-		0	-	-
D	-	-	34	31	-	-
E	-	-	39	49	-	-
F	-	-	20	26	-	-
**Average**	-	-	**37**	**26**	-	-
Standard deviation	-	-	11	19	-	-
**Bonding Partners**			THR48<br>TRP46<br>ASP47<br>ALA49<br>GLY50	THR48		

Lumen	L1		L2		N1	
Simulation	hydroxyl	keto	hydroxyl	keto	hydroxyl	keto
A	24	-	16	-	52	-
B	64	-	14	-	30	-
C	93	-	23	-	49	-
D	82	-	11	-	46	-
E	60	-	10	-	0	-
F	45	-	28	-	25	-
**Average**	**61**	-	**17**	-	**30**	-
Standard deviation	25	-	7	-	20	-
**Bonding Partners**	GLN197<br>PRO205<br>ASN208<br>LEU209		ALA95<br>GLY78<br>TRP97<br>CYS79<br>PHE98		TYR112	

Supplementary Table 3. Hydrogend bond network between astaxanthin and LHCII protein. In the table the H-bond (HB) occupancy is reported, defined as the percentage of time that the H-bond is present between two partner atoms, over the total time of the simulation, excluding from the analysis the first 400 ns of simulation, similarly as in Ref.[5]. The analyses were performed for each simulation (A to F, see Methods). The Hbond plugin of VMD2 was used for the analysis over the atoms of the whole astaxanthin molecules (in site L1, L2 and N1) for binding partners amongst any LHCII residue using the following criteria: Donor-Acceptor distance should be less than 3.5 Å (cut-off distance), Donor-H-Acceptor angle should be less than 30^0 (cut-off angle) and an HB occupancy higher than 10% should be present at least in four simulations. Results of the analysis for the stromal and lumenal sides of the membrane are reported respectively in the green and orange table and are indicated separately for the hydroxyl and keto groups of the carotenoid. The values of HB occupancy (% of the total simulated time after the first 400 ns), the average value (over the simulations) and the standard deviation associated to the average (%) are given per each simulation. At the bottom of each section the residues involved in the H-bond with the hydroxyl or keto group of each carotenoid are listed.

This analysis also shows that astaxanthin bind to the N1 site with HB occupancy values (30 ± 20 %) similar to neoxanthin in wildtype LHCII (44 ± 30 %)[5]. Indeed, while in LHCs associated to photosystem II N1 is generally occupied by a neoxanthin, in a 9-cis-configuration[6,7], recent high-resolution crystal structures of LHCs associated to photosystem I[8,9] revealed that also other Car species can bind in the N1 site and in an all-trans configuration. Via this set of molecular dynamics simulations of LHC-Asta, we confirm that an all-trans astaxanthin can occupy the N1 site of LHCII.

Supplementary references

(1) Croce, R.; Cinque, G.; Holzwarth, A. R.; Bassi, R. The Soret Absorption Properties of Carotenoids and Chlorophylls in Antenna Complexes of Higher Plants. *Photosynth. Res.* **2000**, *64* (2–3), 221–231.

(2) Gradinaru, C. C.; Kennis, J. T. M.; Papagiannakis, E.; van Stokkum, I. H. M.; Cogdell, R. J.; Fleming, G. R.; Niederman, R. A.; van Grondelle, R. An Unusual Pathway of Excitation Energy Deactivation in Carotenoids: Singlet-to-Triplet Conversion on an Ultrafast Timescale in a Photosynthetic Antenna. *Proc. Natl. Acad. Sci.* **2001**, *98* (5), 2364–2369.

(3) Papagiannakis, E.; Kennis, J. T. M.; van Stokkum, I. H. M.; Cogdell, R. J.; van Grondelle, R. An Alternative Carotenoid-to-Bacteriochlorophyll Energy Transfer Pathway in Photosynthetic Light Harvesting. *Proc. Natl. Acad. Sci.* **2002**, *99* (9), 6017–6022.

(4) van Oort, B.; van Grondelle, R.; van Stokkum, I. H. M. A Hidden State in Light-Harvesting Complex II Revealed By Multipulse Spectroscopy. *J. Phys. Chem. B* **2015**.

(5) Liguori, N.; Periole, X.; Marrink, S. J.; Croce, R. From Light-Harvesting to Photoprotection: Structural Basis of the Dynamic Switch of the Major Antenna Complex of Plants (LHCII). *Sci. Rep.* **2015**, *5*, 15661.

(6) Wei, X.; Su, X.; Cao, P.; Liu, X.; Chang, W.; Li, M.; Zhang, X.; Liu, Z. Structure of Spinach

Photosystem II–LHCII Supercomplex at 3.2 Å Resolution. *Nature* **2016**, *534* (7605), 69–74.
(7) Pan, X.; Li, M.; Wan, T.; Wang, L.; Jia, C.; Hou, Z.; Zhao, X.; Zhang, J.; Chang, W. Structural Insights into Energy Regulation of Light-Harvesting Complex CP29 from Spinach. *Nat. Struct. Mol. Biol.* **2011**, *18* (3), 309–315.
(8) Qin, X.; Suga, M.; Kuang, T.; Shen, J.-R. Structural Basis for Energy Transfer Pathways in the Plant PSI-LHCI Supercomplex. *Science (80-.).* **2015**, *348* (6238), 989–995.
(9) Mazor, Y.; Borovikova, A.; Nelson, N. The Structure of Plant Photosystem I Super-Complex at 2.8 Å Resolution. *Elife* **2015**, *4*, e07433.
